# A Tibial Tubercle Fracture Masquerading as an Inferior Pole of the Patella Fracture

**DOI:** 10.5435/JAAOSGlobal-D-22-00029

**Published:** 2023-01-20

**Authors:** E. Graham Englert, Muhammad Waheed, Ehab Saleh

**Affiliations:** From the Beaumont Health Royal Oak, Royal Oak, MI (Dr. Englert and Dr. Saleh), and Oakland University School of Medicine, Rochester, MI (Dr. Waheed).

## Abstract

Pediatric tibial tubercle fractures are uncommon injuries that most often occur in adolescent men. Patients will typically present with anterior knee pain with or without patella alta. This case report describes a tibial tubercle fracture in a 13-year-old man misidentified as an inferior pole patella sleeve fracture on physical examination and preoperative radiographs. The tubercle reduction was secured with cannulated screws while injury to the patellar tendon periosteal sleeve was repaired with suture anchors. This case highlights the utility of advanced imaging when the etiology of extensor mechanism disruption is unclear. Furthermore, it is imperative to set expectations with parents and guardians that the full extent of the injury may only be confirmed under direct visualization in the operating room because of the complexity of such injuries.

The knee is the most commonly injured joint in adolescents. Among knee injuries, tibial tubercle fractures are rare and account for less than 3% of all pediatric fractures and <1% of physeal injuries.^1,2^ Adolescent men performing jumping activities are at an increased risk.^3^ As the proximal tibial physis closes from posterior to anterior and proximal to distal, the tibial tubercle is the last area to fuse thus predisposing it to failure under tensile stress before physiologic epiphysiodesis. Patients with tibial tubercle avulsions will typically present with large knee effusions, anterior knee tenderness, weakened or absent extensor mechanism function, and patella alta depending on the amount of displacement.^3,4^

To guide treatment, the Ogden classification was derived from the Watson-Jones classification system (Figure 1).^3,5,6^ Nonsurgical management in a knee immobilizer or long leg cast is reserved for patients with type I and II fractures, intact extensor mechanism, and adequate fracture reduction (<2 mm of residual displacement). Eighty-eight percent of tibial tubercle avulsion fractures require open reduction and internal fixation.^2,3^

**Figure 1:**
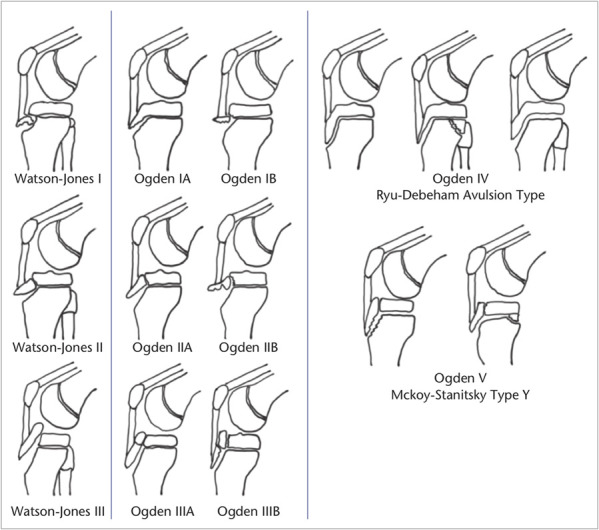
Diagram showing Watson-Jones and Ogden classification systems for tibial tubercle fractures with the Ryu-Debeham and McKoy-Stanitsky modifications (diagram courtesy of Rodriguez et al. 2020 and the British Editorial Society of Bone and Joint Surgery).^18^

The differential diagnosis for anterior knee pain in a child may also include a patella fracture. Although patella fractures account for less than 1% of pediatric fractures, they occur more frequently in adolescent men. Patients commonly present with knee effusion, absent extensor mechanism, and patella alta. Patellar sleeve avulsions are the most common variant and entail separation of the cartilage sleeve that serves as the tendinous attachment to the ossified patella. Radiographs typically reveal patella alta and bony fragments adjacent to the inferior pole. Although nondisplaced fractures with intact extensor mechanisms can be treated nonsurgically in a knee immobilizer or long leg cast, displaced fractures with disrupted extensor mechanisms require surgical intervention.^4^

Identifying the correct etiology for absent extensor mechanism function and patella alta is important for determining the need for surgery and preoperative planning. We present a case in which a tibial tubercle avulsion was initially identified as an inferior pole patella sleeve fracture based on physical examination and radiographs.

## Case Report

**Figure 2:**
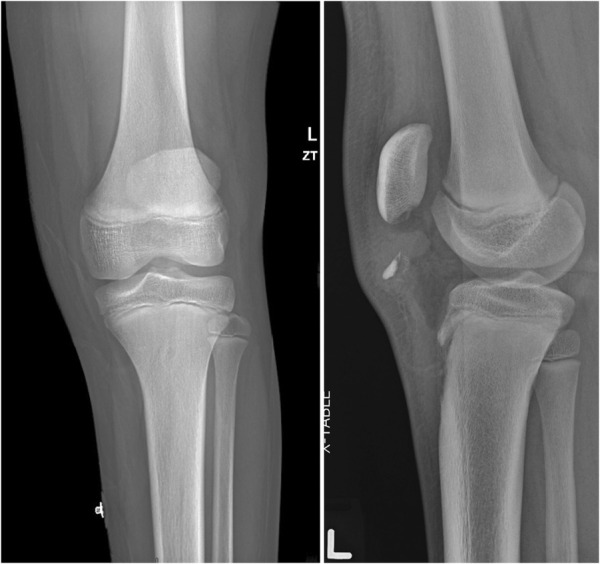
AP and lateral radiographs of the left knee in the Emergency Department.

A 13-year-old man presented to our hospital's emergency department as a transfer from an outside hospital complaining of left knee pain after a fall skateboarding. He was unable to bear weight after the accident and denied antecedent knee pain. The knee was swollen and patella alta was noted on examination. The patient was tender to palpation over the patellar tendon. His extensor function was absent. His medical history was only notable for epilepsy. Knee radiographs revealed open physes, patella alta, and a 13 × 5 mm ossific fragment 13.5 mm distal to the patella (Figure 2). A knee immobilizer was subsequently applied.

**Figure 3:**
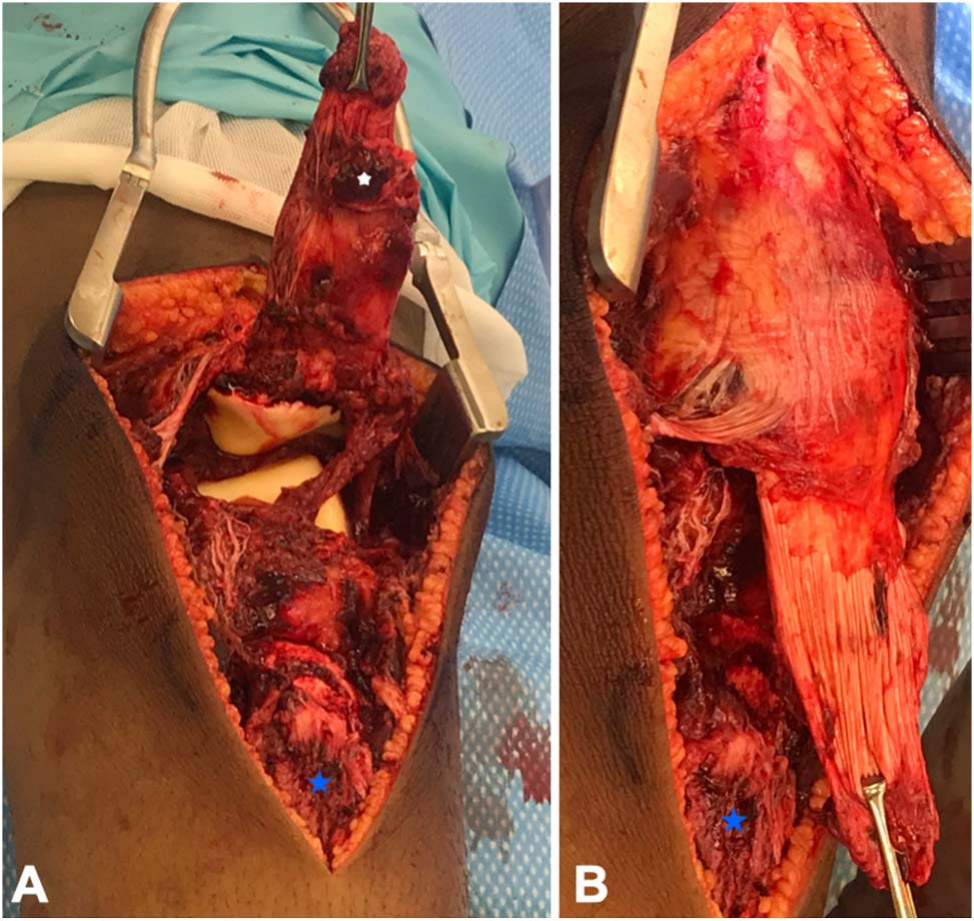
Surgical photographs demonstrating intact patellar tendon, tibial tubercle fragment (white star), patellar tendon sleeve avulsion (held by grasper), and anatomic location for tubercle fragment (blue star).

The patient was brought to the OR and placed in a supine position. A midline incision was made from two fingerbreadths superior to the patella to the level of the tibial tubercle for a presumed open reduction and internal fixation of a patella sleeve fracture. After subcutaneous dissection, a type IB tibial tubercle fracture with an associated patellar tendon periosteal sleeve avulsion from the area distal to the fractured tubercle was identified. The patellar tendon with the tibial tubercle fragment was folded back on itself which gave the appearance of an inferior pole patella sleeve fracture on initial radiographs. There was also disruption of the medial and lateral retinaculum and a small cortical avulsion fracture off the inferior patella without disruption of the cartilage or patellar tendon (Figure 3).

**Figure 4:**
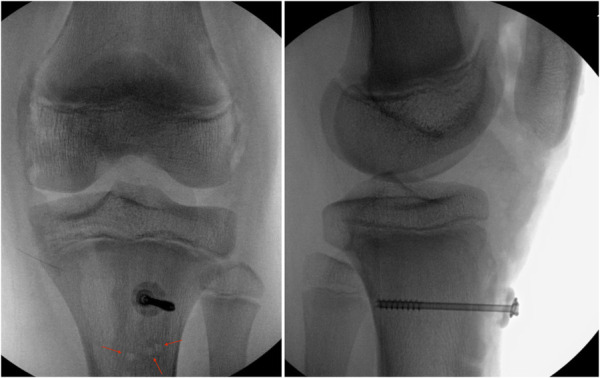
Fluoroscopy of the left knee in the operating room after reduction of the tibial tubercle and placement of a cannulated screw with a spiked washer. The approximate location of the suture anchors are marked with red arrows.

After extending the incision distally and preparing the fracture site, a Stryker Asnis 4.0 partially threaded cannulated screw (Kalamazoo, MI) was used to secure the tibial tubercle reduction. Distal to the tubercle fracture, the periosteal sleeve avulsion was repaired with three 3.0 Arthrex SutureTak suture anchors (Naples, FL) (Figure 4). Finally, the medial and lateral retinaculum was repaired with 0 Vicryl sutures. A bivalved long leg cast was applied with the knee extended.

At 2 weeks postoperatively, his cast was changed and he was permitted to weight-bear as tolerated in his cast with cast shoe. At 6 weeks postoperatively, the patient was placed into a hinged knee brace locked from 0 to 90° with instructions to increase flexion by 10° each week with physical therapy. His knee brace was discontinued at 12 weeks postoperatively. By 20 weeks postoperatively, the patient was ambulating without a limp or discomfort and had full active and passive knee extension. His knee flexion was measured to be 118° actively and 123° passively.

## Discussion

Tibial tubercle fractures with associated patellar tendon periosteal sleeve avulsions are reported infrequently in the literature.^7,8,9,10,11,12^ When such injuries have occurred, standard of care has been screw fixation of the tibial tubercle with transosseous suture or fiber wire repair of the avulsed tendon periosteal sleeve, followed by prolonged immobilization in full extension.^8,9^ In reported cases, preoperative radiographs have demonstrated displaced inferior patella sleeve fragments in close proximity to the tibial tubercle. Our case was unique because of the proximity of the tibia tubercle fragment to the inferior pole of the patella with an associated cortical avulsion fracture off the inferior patella. In 2014, Cash et al. described a 14-year-old man with both an inferior pole patella sleeve injury and tibial tubercle avulsion fracture. Although they repaired their patellar sleeve fracture with a single small fragment screw, this was not necessary in our patient because of the small size of the fragment and continuity of the extensor mechanism at that anatomic location.^13^ In 2021, Sidharthan et al. presented a series of five patients with similar bifocal patellar tendon avulsion fractures after injuries entailing a forceful eccentric contracture of the quadriceps. All five were treated with suture anchor fixation of the avulsed periosteal sleeve in a manner similar to our patient, and all but one of the patients in this series achieved full extension by 7 months postoperatively. The authors of this review emphasized the importance of obtaining advanced imaging to better understand the full extent of injury in patients with traumatic patella alta.^14^ Unfortunately, advanced imaging was not obtained for our patient which would have permitted the correct diagnosis to be made.

Dupuis et al. echoed this sentiment in their retrospective review of pediatric patients with extensor mechanism injuries. The authors recommend the select use of an MRI as a useful adjunct to better delineate the extent of soft-tissue injury for preoperative planning and discussing prognosis.^15^ A less expensive, albeit significantly more operator dependent, alternative to MRI is ultrasonography which has shown utility in assessing additional soft-tissue anatomy in patients with extensor mechanism injuries.^16^ Although the risk of radiation must be considered, Pandya et al.^17^ revealed the utility of CT scan for characterizing fracture morphology and intra-articular involvement in pediatric patients with tibial tubercle fractures.

Pediatric extensor mechanisms injuries are rare, and it is not always evident what structures are disrupted on physical examination. When managing pediatric extensor mechanism injuries, we recommend that surgeons have a low threshold to obtain advanced imaging before surgery if any doubt exists regarding the patient's diagnosis. Furthermore, it is imperative to set expectations with parents and guardians that the full extent of the injury may only be confirmed under direct visualization in the operating room because of the complexity of such injuries.
